# Impact of sarcopenia and sagittal parameters on the residual back pain after percutaneous vertebroplasty in patients with osteoporotic vertebral compression fracture

**DOI:** 10.1186/s13018-022-03009-4

**Published:** 2022-02-20

**Authors:** Jiashen Bo, Xuan Zhao, Zijian Hua, Jia Li, Xiangbei Qi, Yong Shen

**Affiliations:** 1 Department of Orthopaedic Surgery, The Third Hospital of Hebei Medical University, Shijiazhuang, 050051 People’s Republic of China; 2 The Key Laboratory of Orthopedic Biomechanics of Hebei Province, The Third Hospital of Hebei Medical University, 139 Ziqiang Road, Shijiazhuang, 050051 People’s Republic of China

**Keywords:** Sarcopenia, Spinal sagittal parameters, Residual back pain, Osteoporotic vertebral fracture, Percutaneous vertebroplasty, Skeletal muscle mass index

## Abstract

**Objective:**

The objective of this study was to explore the impact of sarcopenia and sagittal parameters on the residual back pain (RBP) after percutaneous vertebroplasty (PVP) for treatment of osteoporotic vertebral compression fracture (OVCF).

**Methods:**

This retrospective study included elderly patients (age range 60–90 years) with OVCF treated with PVP from January 2015 and December 2020 in our hospital. The skeletal muscle mass index (SMI) was calculated by dividing the T12 pedicle level muscle cross-sectional area by the square of body height from chest CT to diagnose sarcopenia. The radiological parameters for measuring the sagittal alignment were included: C7-sagittal vertical axis (SVA), T1 pelvic angle (TPA), lumbar lordosis (LL), thoracic kyphosis (TK), pelvic tilt (PT), sacral slope (SS), pelvic incidence (PI).

**Result:**

According to whether the VAS score > 4, patients were divided into RBP group (56 patients) and Control group (100 patients). There was no difference in age, gender, body mass index, BMD, surgical segment, bone cement usage between the groups (*P* > 0.05). The SMI in RBP group (27.3 ± 5.1) was significantly lower compared to that in Control group (36.8 ± 3.2) (*P* < 0.05). Sarcopenia was present in 19 patients (20.3%) in RBP group, which was significantly more than that in Control group (*P* < 0.05). C7-SVA and TPA was significantly larger in the RBP group than in the Control group (*P* < 0.05). PI and LL was significantly smaller in the RBP group compared to the Control group (*P* < 0.05). However, no significant differences between the two groups with respect to TK, SS and PT (*P* > 0.05).

**Conclusion:**

Poor sagittal parameters and sarcopenia in OVCF patients after PVP were more prone to residual back pain. Larger C7-SVA, TPA and PI-LL mismatch could increase the incidence of RBP in elderly patients with single-segment osteoporotic compression fractures.

## Introduction

Sarcopenia is characterized as a significant loss of skeletal muscle mass and strength, which always correlates with the development of osteoporosis [1–3]. Osteoporotic vertebral compression fracture (OVCF) decreases the vertebral height, increases spinal deformity, and leads to spinal sagittal imbalance [4–6]. Percutaneous vertebroplasty (PVP) has the advantages of pain relief and is widely used [7–9]. Owing to disc and ligament degeneration, elderly patients with osteoporosis often have altered spinal mechanical distribution and reduced paravertebral muscle strength. Patients with OVCF exhibit significant anterior sagittal imbalance compared with healthy adults. In patients with OVCF, PVP is an effective minimally invasive procedure that not only relieves fracture-related pain, but also improves the sagittal balance of the spine by improving local kyphosis.

However, several scholars were still sceptical about the analgesic effect of PVP [10, 11]. Some patients have varying degrees of pain in the postoperative period of PVP, and the postoperative pain of patients is the result of the combined effect of many factors. Previous studies suggested the pains were related to the patient's bone density, cement leakage, compression of the spinal cord and/or nerve roots by cement leakage, the volume of cement injection, postoperative vertebral infection, new fractures, loosening of the bone-cement interface or bone discontinuity, and regular postoperative anti-osteoporosis therapy. In addition, lumbar dorsal fascia injury has also been suggested as a possible factor [12–15]. The objective of this study was to explore the impact of sarcopenia and sagittal parameters on residual back pain (RBP) after PVP, providing theoretical basis for clinical prevention of residual low back pain after PVP.

## Method

This retrospective study analyzed 638 patients with OVCF who underwent PVP in the authors’ institution between January 2015 and December 2020. Inclusion criteria: (i) patients diagnosed with OVCF; (ii) treated with PVP; (iii) follow-up period of more than 12 months. Exclusion criteria: (i) a history of previous spine surgery, (ii) intraoperative leakage of bone cement into the spinal canal and compression of the spinal cord and/or nerve roots, (iii) new postoperative vertebral fracture or postoperative infection, (iv) combined with malignant tumors of the spine, (v) combined with severe cardiac and pulmonary dysfunction, unable to tolerate surgery. This study protocol was approved by the Institutional Review Board of the Third Hospital of Hebei Medical University. All patients signed written informed consent. The clinical procedures were carried out according to the principles in the Declaration of Helsinki.

### Data collection and image analysis

Clinical and radiographic data were examined. Demographic data of the patients were collected, including age, gender, skeletal muscle mass index (SMI), body mass index (BMI), bone mineral density (BMD), and surgical level. For patients with residual low back pain, thoracic and lumbar MR examinations were performed at the 1-year postoperative outpatient follow-up to rule out a new fracture. The Visual Analogue Scale (VAS) was used to evaluate the RBP, with a score of 0 indicating no pain and 10 indicating the most painful pain, and patients were asked to select one of 11 numbers to represent their pain level. The scale is 0–10. In this study, the patients were divided into RBP group (VAS score > 4) and control group (VAS score < 4).

**Fig. 1:**
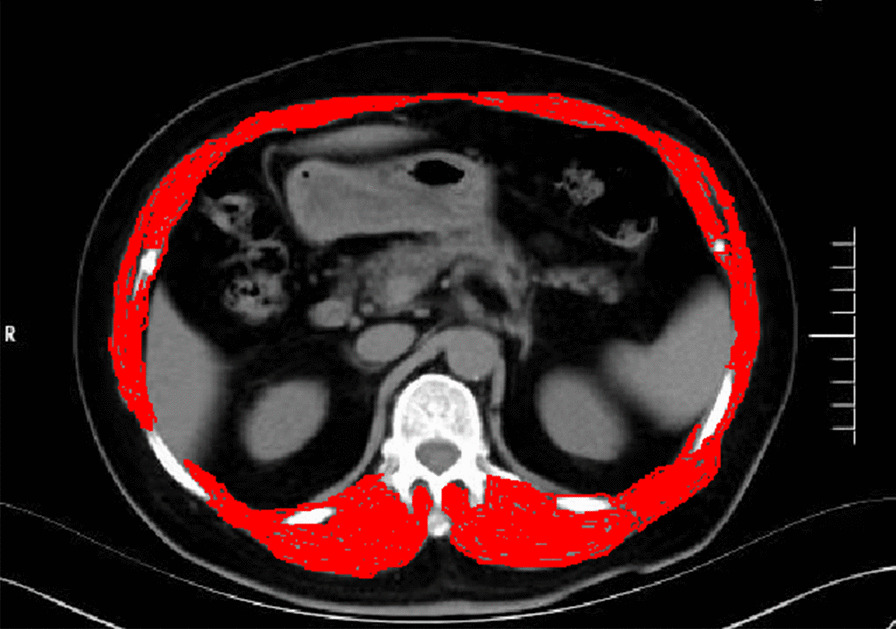
CT images used for the assessment of T12 SMI in OVCF patients. SMI was defined as the sum of the measured muscle area (at the level of the thoracic 12 vertebral body) divided by the square of the patient's height (cm^2^/m^2^). Muscle area was calculated by measuring muscle area including erector spinae, latissimus dorsi, internal abdominal oblique, external abdominal oblique, rectus abdominis, external intercostal muscles and intercostal muscles on CT images

The SMI was calculated from chest CT to diagnose sarcopenia. SMI was defined as the sum of the measured muscle area (at the thoracic 12 vertebral body) divided by the square of the patient's height (cm^2^/m^2^). Muscle area was calculated by measuring muscle area including erector spinae, latissimus dorsi, internal abdominal oblique, external abdominal oblique, rectus abdominis, external intercostal muscles and intercostal muscles on CT images. The images were analyzed using PACS 3.6 software. The diagnostic cut-off values for SMI at the thoracic 12 level were proposed by Nemec et al. [16]. Values below 42.6 cm^2^/m^2^ (male) and 30.6 cm^2^/m^2^ (female) were diagnosed as sarcopenia (Fig. 1).

**Fig. 2:**
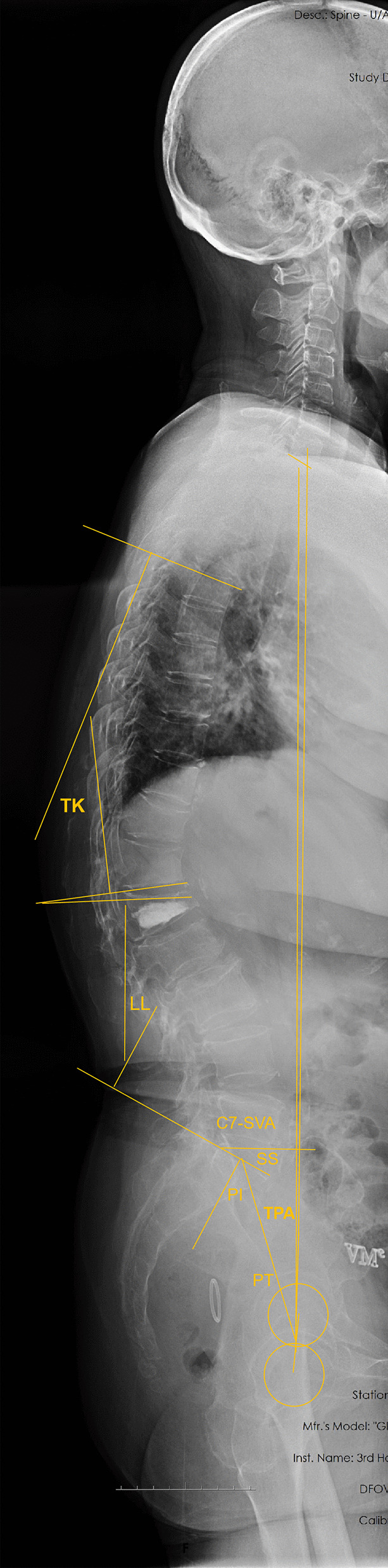
PI: Use the midpoint of the upper endplate of S1 as the vertical line, and the angle between the vertical line and the midpoint of the upper endplate of S1 and the center of the femoral head. If the bilateral femoral heads do not overlap, take two femoral heads The midpoint of the center line is used as the center point. SS: The angle between the upper end plate of S1 and the horizontal line. PT: The angle between the midpoint of the upper endplate of S1 and the center of the femoral head and the vertical line. TPA: The angle formed by the line between the center of the bilateral femoral head and the center of the T1 vertebral body and the line between the center of the femoral head and the midpoint of the S1 superior endplate. C7-SVA: The distance between the C7 plumb line and the upper posterior angle of the sacrum. TK: The angle between the extension line of the upper endplate of T5 and the lower endplate of T12. LL: The angle between the extension line of the upper endplate of L1 and the lower endplate of L5

To analyze the sagittal parameters, the radiographic assessment was performed using full-length radiographs (the radiograms were standardized by full extension of hips and knees). All parameters were measured by two surgeons without patients’ information, and the average value was adopted. The radiological parameters for measuring the sagittal alignment included C7-sagittal vertical axis (SVA), T1 pelvic angle (TPA), lumbar lordosis (LL), thoracic kyphosis (TK), pelvic tilt (PT), sacral slope (SS), pelvic incidence (PI) (Fig. 2).

### Statistical analysis

Statistical analyses were performed with SPSS version 22.0 software. All data were presented as mean ± standard deviation. A *P* value of < 0.05 was considered a statistically significant difference. Parametric statistical analysis was conducted by using the independent *t* test or chi-squared test to identify significant differences between two groups.

## Results

In the present study, a total of 56 patients out of 638 patients with OVCF had poor postoperative back pain relief. These patients were classified as the RBP group (VAS score > 4). Due to the significant difference in the number of cases, 100 patients with significant relief of postoperative back pain were selected using the random number table method to represent the Control group (VAS score < 4). The preoperative VAS score in the Control group (7.3 ± 0.4) was slightly higher than that in the RBP group (7.1 ± 0.5). However, the VAS score at 12 months postoperatively (2.6 ± 1.2) was significantly lower than that in the RBP group (4.3 ± 1.5) (*P* < 0.05).

No significant difference was found in age (*P* = 0.816), gender (*P* = 0.602), BMI (*P* = 0.505), BMD (*P* = 0.125), surgical segment (*P* = 0.461), bone cement usage (*P* = 0.383) between the groups. None of them presented a new fracture or required additional surgery for a new fracture. The SMI in the RBP group (27.3 ± 5.1) was significantly lower compared with that in the Control group (36.8 ± 3.2) (*P* < 0.05). Sarcopenia was present in 19 patients (33.9%) in the RBP group, which was significantly more than 21 patients (21.0%) in the other group (*P* < 0.05) (Table 1).

**Table 1:** Comparison of demographic characteristics between the RBP group and control group

	RBP group	Control group	*P* value
Age (years)	70.2 ± 6.3	69.5 ± 5.9	0.816
Gender (M/F)	21/35	33/67	0.602
BMI (kg/m^2^)	25.8 ± 1.5	26.3 ± 1.8	0.505
BMD (T-score)	3.1 ± 1.0	2.9 ± 1.1	0.125
*Surgical level*			0.461
T5–9	9	23	
T10–L2	31	46	
L3-5	16	31	
Bone cement usage (ml)	5.1 ± 0.5	5.0 ± 0.6	0.383
SMI	27.3 ± 5.1	36.8 ± 3.2	0.000
*Sarcopenia*			0.000
Yes	19	21	
No	37	79	

Regarding the global sagittal parameters, C7-SVA was significantly greater in the RBP group (10.6 ± 8.1 cm) in comparison with the Control group (4.5 ± 3.8 cm) (*P* = 0.000), and TPA was significantly higher in the RBP group (20.8° ± 5.6°) compared to the Control group (11.5° ± 6.3°) (*P* = 0.000). For local sagittal parameters, PI and LL (49.5° ± 15.6°, 26.8° ± 9.5°) were significantly smaller in the RBP group than in the Control group (52.6° ± 11.8°,39.2° ± 7.3°), and PI-LL was significantly higher in the RBP group (23.3° ± 9.3°) than in the Control group (13.5° ± 8.8°) (*P* = 0.000). However, no significant difference was found between the two groups with respect to TK, SS and PT. (*P* > 0.05) (Table 2).

**Table 2:** Comparison of sagittal parameters of patients after PVP between the RBP group and control group at the last follow-up

	RBP group	Control group	*P* value
C7-SVA (cm)	10.6 ± 8.1	4.5 ± 3.8	0.000
TPA (°)	20.8 ± 5.6	11.5 ± 6.3	0.000
TK (°)	32.1 ± 12.5	35.5 ± 10.8	0.105
TLK (°)	10.9 ± 5.1	12.5 ± 3.6	0.479
LL (°)	26.8 ± 9.5	39.2 ± 7.3	0.000
PI (°)	49.5 ± 15.6	52.6 ± 11.8	0.038
PT (°)	22.8 ± 9.5	21.2 ± 9.3	0.469
SS (°)	27.1 ± 8.6	30.9 ± 6.5	0.332
PI-LL (°)	23.3 ± 9.3	13.5 ± 8.8	0.000

## Discussion

The clinical outcomes of PVP are affected by multiple factors. Among the patients included in this study, no significant difference was found in age, gender, BMI, BMD, surgical segment, and bone cement dosage between the RBP group and the Control group. The result demonstrated that sarcopenia and sagittal balance have a more significant impact on residual pain after PVP.

The human body maintains a relatively stable posture with minimal energy consumption, to reduce the impact of the spine and spinal cord when standing or exercising. Sagittal balance of the trunk is mainly determined by the alignment of the spine and pelvis, which is essential to maintain normal spinal biomechanics. If the balance of the spine is disrupted, the human body needs to exert more strength to stay upright, leading to fatigue and pain [17–19]. C7-SVA is an important parameter reflecting the global spinal sagittal balance. Previous studies suggested that the C7-SVA in OVCF patients is greater than that in healthy people. The compression of the fractured vertebral body and the aggravation of the kyphosis deformity lead to the forward movement of the body's center of gravity and the increase of C7-SVA [20–22]. TPA reflects the global and local spine-pelvic sagittal balance and is closely related to the patient's quality of life. Schwab and Protopsaltis et al. [23, 24] analyzed the influence of radiographic parameters on the clinical symptoms in adult degenerative scoliosis. Compared with C7-SVA, TPA has a higher correlation with clinical symptoms. The current study found the TPA and C7-SVA in the RBP group was greater than the Control group. Compared with the Control group, the RBP group showed more imbalance in the sagittal plane.

The change of pelvic position is considered an essential role in the compensation of spinal imbalance. The occurrence of OVCF leads to LL decrease and C7-SVA increase, resulting in trunk tilts forward. Simultaneously, spinal balance is maintained by the compensatory posterior pelvic rotation followed by the corresponding changes in hip and knee joints. Once spinal kyphosis and hip degeneration in elderly patients exceed the capacity of the compensation mechanism, the sagittal imbalance will occur [25–27]. The loss of LL is one of the initiating factors of the compensation mechanism in patients with sagittal imbalance. PI reflects the compensatory ability in maintaining overall spinal balance and reducing the forward tilt of the trunk. To obtain the balance between the spine and the pelvis, humans will use various compensatory mechanisms to pull the trunk backward, such as reducing thoracic kyphosis (decreasing TK), pelvic tilting (increasing PT), etc., thereby pulling back the center of gravity [28–30]. The smaller the LL, the larger the PI-LL, and the greater the degree of mismatch between the spine and pelvis. In this study, the PI and LL in the Control group were significantly greater than those of the RBP group, and PI-LL was the opposite (all *P* < 0.05). Patients in the RBP group had smaller PI and LL, larger PI-LL mismatch, which means the weaker the ability to compensate for the imbalance in the sagittal plane (increased SVA and TPA), the greater the degree of spinal deformity, and more likely it to cause low back pain.

Elderly adults usually suffer from spinal arthritis, degenerative disc disease, decreased bone density and sarcopenia. Sarcopenia is a progressive and extensive skeletal muscle disease involving the decrease of muscle mass and function that is associated with increased adverse outcomes. The pathophysiology of sarcopenia is multifactorial, seriously affecting the daily behavior ability and life quality of the elderly. Briefly summarized as mechanical factors acting on the mechanical scaffolds and myotubes composed of bone cells; musculoskeletal systems interact with each other to influence the release of chemical factors; crosstalk and fat infiltration factors in muscle and bone at paracrine and endocrine levels lead to decreased muscle strength and increased incidence of fractures; nutritional deficiency accelerates bone loss and reduces muscle protein synthesis; decreased individual exercise and decreased neuro-muscular function indirectly affect muscle and bone anabolism [31]. According to the bowstring principle, in the process of spinal compensation, muscles act as tension bands. The better the muscle quality, the stronger the compensatory ability of the spine [32]. Iolascon et al. studies of 67 female patients with vertebral fractures found that 35 cases had a fracture of one vertebral body, the incidence rate was 52.23%, and 8 cases (22.85%) had reduced skeletal muscle mass. The remaining 32 cases had multiple vertebral fractures, the incidence rate was 47.76%, of which 14 cases (43.75%) had reduced skeletal muscle mass [33]. DiMonaco et al. study of 313 elderly women with hip fractures found that 180 patients had reduced skeletal muscle mass, with an incidence rate of 58% [34]. The above-mentioned literature suggested that the reduction of skeletal muscle mass may be related to OVCF. In addition to increasing the risk of falls or trauma in elderly patients, sarcopenia combined with osteoporosis often has a more negative impact on the treatment and recovery of fractures. The present study found that the SMI in the RBP group was much lower than that the Control group, indicating that patients with sarcopenia improved poorly in postoperative back pain symptoms.

Sarcopenia has significant deleterious effects on muscle strength and balance, increases the incidence of osteoporotic fracture-related complications. Therefore, patients with sarcopenia are at higher risk of falls, especially those with osteopenia, which is a major cause of fractures and can lead to poor postoperative outcomes. Chen et al. analyzed patients with hip fracture after surgery experience a significant loss of muscle mass and may be detrimental to functional recovery among geriatric patients undergoing surgery. They emphasized a potential treatment target of maintaining muscle mass to improve prognosis in patients with sarcopenia [35]. For OVCF patients with sarcopenia, besides the treatment of the fracture, the sarcopenia also requires intervention and treatment at the same time. The perioperative period can be combined with rehabilitation and nutrition, such as early nutritional intervention, supplementing appropriate amounts of protein, essential amino acids, and fatty acids, etc., and symptomatic treatment such as pain relief if necessary. During rehabilitation training, the doctor should guide the patient and emphasize to the patient to avoid falling again during the exercise. The focus of treatment is to improve the muscle mass, strength, and general condition. It should be noted that clinicians usually associate sarcopenia with thinness. However, obese patients also develop sarcopenia. If only obesity is treated, it may lead to undesirable consequences.

There were several limitations in the present study. First, although this study used strict inclusion criteria and excluded the interference of age, gender, bone density, bone cement leakage, number of injured vertebrae and surgical approach, it was only a retrospective study, and the sample size of poor postoperative low back pain relief was small. Second, this study did not include patients after kyphoplasty, which has certain shortcomings. Anti-osteoporosis treatment after PVP in OVCF patients is important for pain symptom relief. Third, in this study, only preoperative BMD T values were analyzed, and the effect of anti-osteoporosis treatment was not considered. Forth, the effects of vertebral height restoration and cemented disc leakage after PVP treatment were not analyzed. Therefore, a prospective, controlled study is needed to further investigate the quantitative effects of these factors on residual pain after surgery.

## Conclusion

In summary, poor sagittal balance and sarcopenia in OVCF patients after PVP were more prone to residual back pain. Larger C7-SVA, TPA and PI-LL mismatch could increase the incidence of low back pain in elderly patients with OVCF. For patients with abnormal sagittal parameters and sarcopenia, appropriate lengthening of bedtime, wearing a brace and systematic functional exercise can be recommended.

## Data Availability

Not applicable.

## Abbreviations

OVCF: Osteoporotic vertebral compression fracture
PVP: Percutaneous vertebroplasty
RBP: Residual back pain
SMI: Skeletal muscle mass index
BMI: Body mass index
BMD: Bone mineral density
VAS: Visual Analogue Scale
SVA: C7-sagittal vertical axis
TPA: T1 pelvic angle
LL: Lumbar lordosis
TK: Thoracic kyphosis
PT: Pelvic tilt
SS: Sacral slope
PI: Pelvic incidence
